# *FibH* Gene Complete Sequences (FibHome) Revealed Silkworm Pedigree

**DOI:** 10.3390/insects14030244

**Published:** 2023-02-28

**Authors:** Wei Lu, Tong Zhang, Quan Zhang, Na Zhang, Ling Jia, Sanyuan Ma, Qingyou Xia

**Affiliations:** 1 State Key Laboratory of Silkworm Genome Biology, Southwest University, Chongqing 400715, China; 2 Biological Science Research Center, Southwest University, Chongqing 400715, China; 3 Integrative Science Center of Gerplasm Greation in Western China (CHONGQING) Science City & Southwest University, Chongqing 400715, China

**Keywords:** pan-genome, *FibH* gene, FibHome, cis-regulatory element, silkworm pedigree

## Abstract

**Simple Summary:**

The fibroin heavy chain (*FibH*) is the core protein of silkworm silk, and a systematic analysis of its sequence (primary structure) is essential for understanding silkworm silk fibers. However, only a few *FibH* sequences have been identified. The recently published high-resolution silkworm pan-genome was useful in analyzing the *FibH* gene systematically. In this study, we extracted the complete *FibH* sequences of wild silkworms, local and improved strains from the silkworm pan-genome, bringing the total number of known silkworm *FibH* genes to 286. We characterized in detail the *FibH* variation during domestication and breeding and established a silkworm pedigree based on the *FibH* gene sequence. Additionally, we investigated the relationship between the cis-regulatory elements of *FibH* gene and silk yield in wild and domesticated silkworms. Our study laid the foundation for artificial silk as well as provided guidance for silkworm breeding.

**Abstract:**

The highly repetitive and variable fibroin heavy chain (*FibH*) gene can be used as a silkworm identification; however, only a few complete *FibH* sequences are known. In this study, we extracted and examined 264 *FibH* gene complete sequences (FibHome) from a high-resolution silkworm pan-genome. The average *FibH* lengths of the wild silkworm, local, and improved strains were 19,698 bp, 16,427 bp, and 15,795 bp, respectively. All *FibH* sequences had a conserved 5′ and 3′ terminal non-repetitive (5′ and 3′ TNR, 99.74% and 99.99% identity, respectively) sequence and a variable repetitive core (RC). The RCs differed greatly, but they all shared the same motif. During domestication or breeding, the *FibH* gene mutated with hexanucleotide (GGTGCT) as the core unit. Numerous variations existed that were not unique to wild and domesticated silkworms. However, the transcriptional factor binding sites, such as fibroin modulator-binding protein, were highly conserved and had 100% identity in the *FibH* gene’s intron and upstream sequences. The local and improved strains with the same *FibH* gene were divided into four families using this gene as a marker. Family I contained a maximum of 62 strains with the optional *FibH* (Opti-*FibH*, 15,960 bp) gene. This study provides new insights into *FibH* variations and silkworm breeding.

## 1. Introduction

*Bombyx mori*, the domesticated silkworm, is an economically important insect that has played a vital role in human economic and cultural history. According to evolutionary analysis and archeological evidence, domesticated silkworms originated in the middle and lower reaches of the Yellow River and were domesticated from Chinese wild silkworms [1,2,3]. The Chinese trimolter may have been the first domesticated local strain. Following selection and dissemination, the trimolter silkworm gradually evolved into a Chinese tetramolter strain, a European strain, and a tropical variety [4]. Local strains were bred into improved strains, including Chinese and Japanese improved strains to improve silk quality and yield. The Chinese improved strains were chosen from the Chinese tetramolter strains, whereas the Japanese improved strains were derived primarily from European strains [4]. Evolutionary analysis based on conserved genes revealed the origin of silkworms but could not reveal the silkworm breeding history. With the exception of the breeding process of some improved strains, the pedigree of local and some improved strains has remained unclear.

Silkworm silk is mainly composed of three fibroin and five sericin proteins, with the fibroin heavy chain (FibH), encoded by the *FibH* gene, having the highest content and molecular weight [5,6,7,8]. The first reported *FibH* gene is 16,788 bp long, consisting of two exons (67 bp, 15,750 bp) and one intron. Exon II comprises non-repetitive sequences at both ends and numerous tandem repeats [9,10]. We previously obtained the complete *FibH* sequences of seven wild (*Bombyx mandarina*) and seventeen domesticated silkworms using CACS method [11]. Their exon I is identical, the non-repetitive sequences at both ends of exon II are very conservative, and the introns are dominated by single-base mutations. The repetitive sequence of exon II is composed of the same repeat units, but their number and arrangement order differ, resulting in considerable differences in the sequence and length of the *FibH* gene between wild and domesticated silkworms. Wild silkworms had the longest average *FibH* and the most variation, followed by local strains, whereas improved strains had the shortest average *FibH*, and the fewest variations. Wild silkworms and local strains do not share the same *FibH* gene. This gene is identical in all improved strains, except for two mutants (grr and nds), which are closely related to their breeding history. The parents of strain 872 were strains 46 and 782, the grandfathers were strains 794 and 796, and the parents of strain 54A were strains 532 and 794, implying that the *FibH* gene could be used as a marker gene for identifying silkworm pedigree.

There are thousands of silkworm strains, including wild silkworms, local and improved strains, and genetic mutants, but only 25 strains have known *FibH* sequences [9,12,13]. Tong et al. recently established a high-resolution pan-genomic database using deeply re-sequencing and long-read sequencing [3]. In this study, we extracted *FibH* sequences from the silkworm pan-genome, characterized the *FibH* variation, and developed a silkworm pedigree. To investigate the relationship between cis-regulatory elements and silk yield, the intron, upstream, and downstream sequences of the *FibH* gene were examined. Our results improve our understanding of the *FibH* gene in addition to providing insights for silkworm breeding.

## 2. Materials and Methods

### 2.1. Data Source

Tong et al. assembled 545 high-resolution silkworm genomes and deposited them in the China National GeneBank DataBase (CNGBdb; https://db.cngb.org/ (accessed on 10 November 2022)) [3]. Sample IDs (such as BomW2, BomL1, and BomP18) in Supplementary Table S2 were used to retrieve data from the CNGBdb. Each sample ID contained the assembled chromosome and contig data, each with a unique assembly number. The assembled chromosome data were downloaded for subsequent analyses.

### 2.2. Sequence Analysis

The complete *FibH* sequence, as well as its upstream and downstream sequences, were extracted from the assembled chromosomal genome using the terminal sequence (20 bp) of the Dazao strain’s *FibH* gene as a barcode. A few *FibH* sequences were investigated and aligned using Bioedit 7.0.9.0 and Jalview 2.11.2.5 [14]. The online software mafft (https://mafft.cbrc.jp/alignment/server/ (accessed on 12 November 2022)) [15] was used to perform multiple sequence alignment of all *FibH* genes and draw a phylogenetic tree based on the average linkage (UPGMA) algorithm. TNR and RC sequences were aligned by online software mafft and their identity was calculated by DNAman version 9.0 (Lynnon Biosoft, San Ramon, CA, USA). RNA22 [16] and RNAhybrid [17] were used to predict miRNA targeting sites existing in the 3′ UTR of all *FibH* genes.

### 2.3. Motif Identification

A novel motif in the *FibH* gene was discovered by MEME in MEME-Suite 5.5.0 [18,19]. The parameters were set to default, except for the number of motifs to be searched (four motifs), the minimum motif width (18 bp), the minimum number of sites per motif (10 sites), and the distribution of any number of repetitions (anr) in *FibH*. Each amorphous region of the *FibH* gene had one *Nde* I site [11], which was used to distinguish the amorphous regions. We used MAST in the MEME-Suite to scan Nde I sites. All the parameters were set to default, except for the motif sequence (restriction site of *Nde* I) and the *FibH* sequence.

### 2.4. Visualization

TBtools software [20] was used to visualize the motifs detected and scanned by MEME and MAST in MEME-Suite. The phylogenetic tree was modified using the online software iTOL (https://itol.embl.de/ (accessed on 1 December 2022)) [21]. The aligned introns as well as the upstream and downstream sequences were visualized through the NCBI Multiple Sequence Alignment Viewer 1.22.2 (https://www.ncbi.nlm.nih.gov/projects/msaviewer/ (accessed on 5 December 2022)). A map was constructed using the software Mark O’Travel 3.2.1 (https://download.cnet.com/Mark-O-Travel-your-travel-map-Where-you-ve-been/3000-20428_4-76133240.html (accessed on 1 December 2022)); the geographical distribution of silkworms was marked on the map by Adobe Illustrator 2021 (Adobe Systems, San Jose, CA, USA).

## 3. Results

### 3.1. FibH Gene of High Quality from the Silkworm Pan-Genome

**Figure 1:**
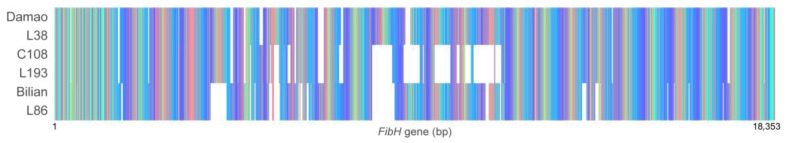
Comparison of the fibroin heavy chain (*FibH*) genes from pan-genome and as obtained by CACS. The *FibH* genes of Damao, C108, and Bilian were obtained by CACS [11], and L38, L86, and L193 were from the pan-genome.

Oxford Nanopore Technologies (ONTs) have long reads and systematic errors that are significantly biased and primarily located in the homopolymer and tandem repetition regions [22]. In a previous study, the *FibH* gene from Cpf1-based enrichment and ONT (CEO)-enriched sequencing (coverage depth 87.07×) lost three bases in polythymine and polyguanine of introns, resulting from sequencing errors [13]. Tong et al. de novo assembled 545 silkworm genomes with average sequencing depth of 97× through long-read sequencing [3]. To assess these genomes, we first extracted *FibH* sequences from the genomes of three local strains: L38 (81×), L86 (72×), and L193 (103×). Compared to the *FibH* of Damao, C108 and Bilian strains obtained by CACS [11], L38 and Damao, L193 and C108, Bilian, and L86 had identical *FibH* gene, indicating that the pan-genome was of high quality and could be used for entire *FibH* analysis (Figure 1, Tables S1 and S2).

### 3.2. FibH Gene Complete Sequences (FibHome) of 264 Silkworms

**Figure 2:**
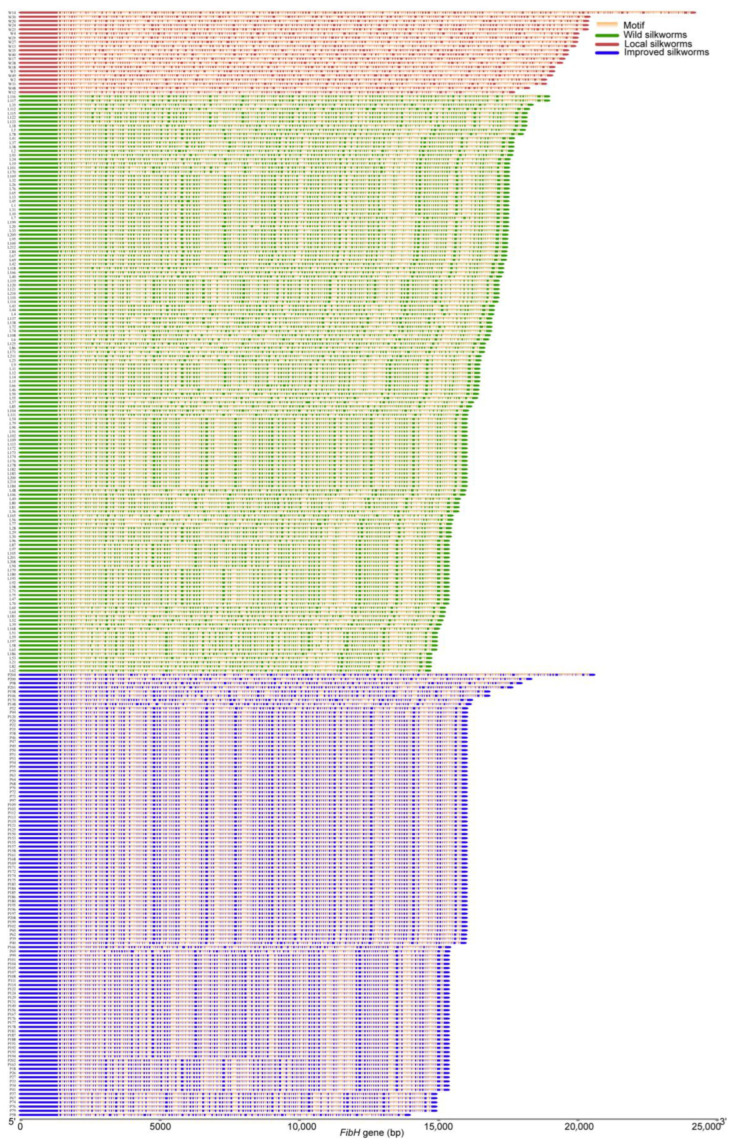
*FibH* gene complete sequences (FibHome) of 264 silkworms from the pan-genome. All *FibH* sequences were unaligned and arranged by length. Motif, orange; Wild silkworms, red; Local silkworms, green; Improved silkworms, blue.

Tong et al. assembled 545 high-resolution silkworm genomes, including 39 wild silkworms, 160 local and 118 improved strains, and 228 genetic stocks [3]. Excluding *FibH* with polyguanine and polyN (N representing any base), we extracted 264 complete *FibH* sequences from the genomes of wild (20), local (138), and improved silkworm strains (106) (Figure 2). All *FibH* genes were composed of the same motif (AGGWRSYGGWGCTGGYTCAGGWGCTGGTGCTGGTKCAGGWGCTGGTGCT, W = T or A, R = G or A, S = G or C, Y = T or C, K = T or G) and were rich in GC (Figure 2). Except for W14, which had 60.0364% GC, the GC content of the other *FibH* sequences ranged from 58.9449% to 59.6878% (Tables S1 and S2). Most of the silkworms in genetic stocks were mutants of unknown origin, so we did not focus on their *FibH* genes.

The length of the *FibH* sequence extracted from wild silkworms varied from 24,162 bp (W14) to 17,664 bp (W12), with an average length of 19,698 bp. The local strains’ *FibH* genes ranged in size from 14 to 19 kb, with an average length of 16,427 bp. *FibH* genes of the same length (15,960 bp) were observed in 11.59% of local strains (L79, L90, L91, L102, etc.). The improved strains had an average *FibH* of 15,795 bp. Except for P204 (20,541 bp), the *FibH* genes of other improved strains were distributed in 13–19 kb. Of the improved strains, 46.23%, 23.58%, 7.55%, and 4.72% had 15,960 bp, 15,318 bp, 15,315 bp, and 14,874 bp *FibH* genes, respectively (Figure 2). These results implied that the *FibH* gene is shortened during domestication.

### 3.3. Conserved Terminal Non-Repetitive and Variable Repetitive Cores of FibH Genes

**Figure 3:**
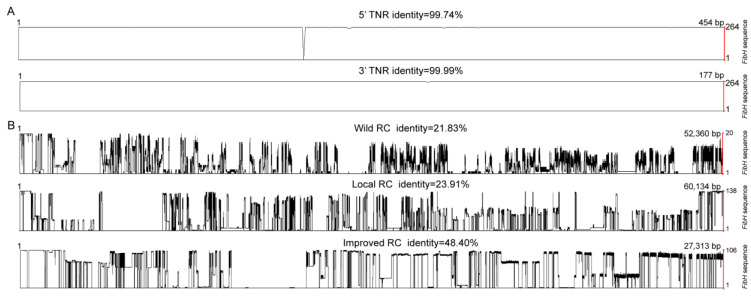
Conserved analysis of the *FibH* gene. (**A**) Similarity analysis of TNR of 264 *FibH* sequences. 5′ or 3′ TNR, 5′ or 3′ terminal non-repetitive. (**B**) Similarity analysis of RC of wild silkworms, local and improved strains. RC, repetitive core.

*FibH* gene coding sequences in wild and domesticated silkworms were composed of 5′ and 3′ terminal non-repetitive (5′ and 3′ TNR, respectively) sequences and a repetitive core (RC). The 3′ TNR of 264 silkworm *FibH* genes from the pan-genome was highly conserved, with 99.99% identity. Except for the 3′ TNR of one improved strain (P204) and two wild silkworms (W17, W35) with base substitutions, the others were the same (Figure 3A). In contrast, the variation of the 5′ TNR was more than that of the 3′ TNR, with 99.74% identity (Figure 3A). One local strain (L96) had a base insertion, resulting in a frameshift mutation and failure of silkworm spinning. This may be due to a sequencing error. Two silkworm strains (L93 and P201) had one base substitution at the same position, and six local silkworm strains had three consecutive base deletions (CAT) at the same position. These variations originate from single nucleotide polymorphisms in the silkworm strains. Some wild silkworms had 1–4 base substitutions, but no insertions or deletions (indels) were identified. The 5′ TNR of the other silkworms was the same (Figure 3A). The high conservation of 5′ and 3′ TNR implied that they may play key role in silk formation.

The RC of *FibH* gene (16.12% identity) was more variable in length and sequence than the 5′ and 3′ TNR, which is consistent with previous studies [11]. The RC of wild silkworms was the longest and each wild silkworm had a unique RC with 21.83% identity (Figure 2). The average RC of the local strains was shorter than that of the wild silkworms and had 23.91% identity. The improved strains had the shortest RC and least difference, with 48.40% identity (Figure 3B). The same RC of 14,319 bp and 13,692 bp was shared by 48.11% and 23.58% of improved silkworms, respectively (Table S3).

### 3.4. FibH Gene Mutated with GGTGCT as the Core Unit

**Figure 4:**
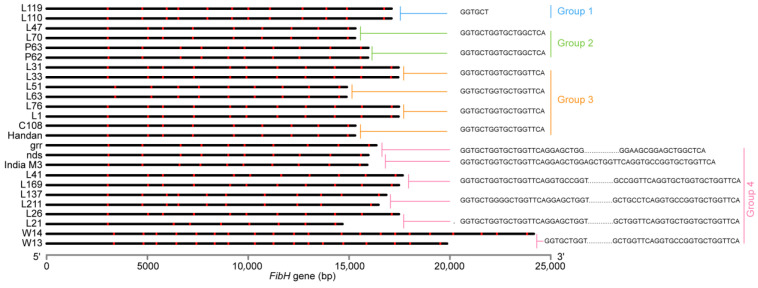
*FibH* gene with differential sequence. The nucleotide sequence is the differential sequence between two adjacent silkworm *FibH* genes. All differential sequences were divided into four groups. The *FibH* gene and the amorphous region identified by *Nde* I are represented by black horizontal lines and red blocks, respectively.

The *FibH* of some silkworms differed from one another in the pan-genome by only one repeat, that is, the differential sequence. All differential sequences were classified into four groups. The differential sequence of Group 1 had only six bases (GGTGCT). The sequence difference between Groups 2 and 3 was 18 bp, and the two groups differed by only one base, and both encoded the hexapeptides GAGAGS, which are the repetitive units of FibH. Group 4 had a 54–4302 bp differential sequence that could encode a complete peptide. The first six bases of the other three groups of differential sequences, with the exception of Group 1, were GGTGCT (encoding dipeptide GA), and the last three bases were TCA (serine) (Figure 4, Table S4). Both dipeptides, GA and serine, are components of the repetitive GAGAGS unit. There were two or more differential sequences containing GGTGCT between *FibH* genes of other silkworms. These findings indicated the *FibH* gene mutated with hexanucleotide GGTGCT as the core unit.

### 3.5. Cis-Regulatory Elements Regulating the FibH Gene Were Highly Conserved

**Figure 5:**
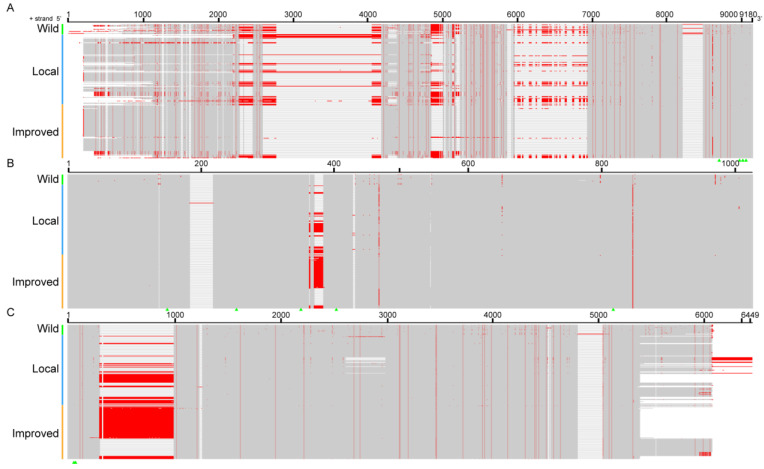
Comparison of the introns and upstream and downstream sequences of the *FibH* gene in wild silkworms, local and improved strains. (**A**–**C**) Upstream, intron, and downstream sequence alignment of the *FibH* gene. Red lines and green triangles indicate sequence differences and cis-regulatory elements, respectively.

The fiber content of FibH, as the main component of cocoon silk, determines the silk yield [23]. The *FibH* gene is highly expressed in the fifth instar larvae and the silkworm’s posterior silk gland and is tightly regulated by cis-regulatory elements (including E, C sites, and TATA-box) and trans-regulatory factors (including Bmdimm, SGFs, FMBPs, Fkh, and POU-M2) [24,25,26,27,28,29]. We found those cis-regulatory elements in the 5 kb upstream sequences of 264 *FibH* genes were highly conserved and had 100% identity (Figure 5A). In addition to the upstream sequence, the intron is also involved in the regulation of the *FibH* gene [24]. The *FibH* gene has only one intron. The L84 intron was 1007 bp long, and the other introns were 958–988 bp long, with 971 bp and 986 bp accounting for 51.14% and 37.50%, respectively. Binding sites for the transcription factor FMBPs were present on all of the silkworm *FibH* introns, suggesting their important role in regulating *FibH* expression (Figure 5B).

There are currently few studies on the downstream regulatory sequence of *FibH*, and it remains unknown if it regulates the *FibH* gene. The 3′ UTR is the first 85 bp of the downstream 5 kb sequence of *FibH*. Except for W35 and L62, all silkworms had the same 3′ UTR. There is an E-box in the downstream sequence of the 3′ UTR that may be involved in regulating the *FibH* gene. Bmo-miR-2739 was the first miRNA discovered in domesticated silkworms, and it can bind to the 3′ UTR to regulate target genes [30,31]. The RNA22 and RNAhybrid predicted target sites existed in the 3′ UTR of all *FibH* genes. There was no specific sequence or variation in the upstream and downstream sequences or introns of *FibH* gene of wild and domesticated silkworms (Figure 5C). These findings suggested that cis-regulatory elements are essential for *FibH* expression, but are not the cause of silk yield differences between silkworm strains, which are likely to be mainly determined by trans-acting factors.

### 3.6. Silkworm Pedigree Revealed by FibHome

**Figure 6:**
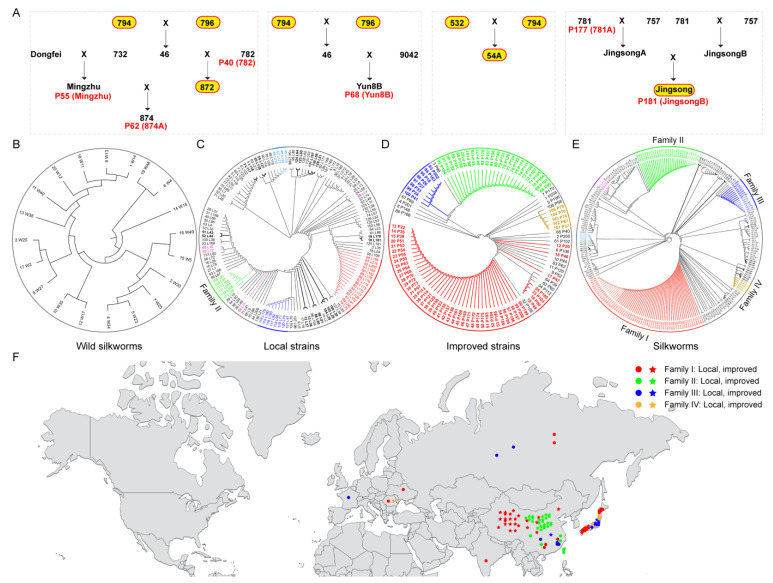
Silkworm pedigree based on FibHome. (**A**) Silkworm family of improved strains. Domesticated silkworms with identical *FibH* genes from a previous study [11], yellow background; domesticated silkworms from the pan-genome, red font. (**B**) The phylogenetic tree of wild silkworms. (**C**) The phylogenetic tree of local strains. (**D**) The phylogenetic tree of improved strains. (**E**) The phylogenetic tree of silkworms containing wild silkworms, local and improved strains. The branches of the same color had the identical *FibH* gene except for the black ones, in which the thickened branches were improved strains. (**F**) Geographical distribution of silkworms in Families I, II, III, and IV. Local strains, circle; Improved strains, Pentacle. Family I, red; Family II, green; Family III, blue; Family IV, yellow.

In a previous study, we observed that improved strains (breeding strains, excluding mutants grr and nds) had identical *FibH* genes [11], which is consistent with their breeding process (Figure 6A). In the pan-genome, strains P181 and P177, P68 and 794, 796 had the same *FibH* gene. P181 (JingsongB) is a binary hybrid of P177 (781A) and 757, while P68 (Yun8B) is a ternary hybrid of strain 794, 796, and 9042, and strain 872 is a ternary hybrid of 794, 796, and 792. Strain 872 (15,960 bp) had the same *FibH* gene as 794 and 796, but differed from P40 (strain 782, 15,920 bp), indicating that the *FibH* gene of strain 872 was derived from strain 794 and 796. These results suggested that *FibH* could be used as a marker gene for silkworm identification.

In the *FibH* sequence extracted by us, not all wild silkworms had the same *FibH* gene. As a result, they could not be clustered (Figure 6B). There were mainly five families of local strains, with a maximum of 16 strains in one family (Figure 6C). There were four improved strain families with 5–47 silkworm strains (Figure 6D). Local and improved strains with identical *FibH* genes were classified into four large clusters: Family I (62 silkworms), II (31 silkworms), III (16 silkworms), and IV (6 silkworms) (Figure 6E and Table 1). The local strains of Families I and III were distributed in many Asian and European countries (mainly China and Japan). The improved strains were all from China and Japan. Both local and improved strains of Family II were obtained from China. Family IV had only one local strain and five improved strains from Romania and Japan (Figure 6F). These results implied that silkworms, particularly local strains, are not confined to one geographic region but spread throughout many countries, with improved strains primarily bred in China and Japan.

## 4. Discussion

FibH is the main component of silkworm silk, and its sequence (primary structure) is the structural basis for the excellent mechanical properties of silk fiber [32,33]. The analysis of its sequence is helpful for understanding the formation mechanism of silk fibers and silk formation. The *FibH* gene has been studied for more than 30 years; however, few complete *FibH* sequences have been reported because of its long sequence (up to 20 kb), rich GC, and repetitive sequences [9,13,34]. Single-molecule sequencing has been used to resolve the complete *FibH* sequence in recent years [3,4], but it is expensive. In this study, we have fully mined the pan-genome data and extracted the *FibH* and its upstream and downstream sequences from wild silkworms, local and improved strains, and some genetic mutants, providing a comprehensive understanding of the *FibH* gene and silkworm breeding.

We attempted to describe the complete sequence of the *FibH* gene and have established the CEO and CACS methods successively [11,13]. The CEO method based on Cpf1 and ONT had an 87.07× coverage depth and 2.29-fold target enrichment. The *FibH* obtained by CEO was identical to that obtained by P50T [11], except that three bases were deleted in the intron. CACS is a cloning and sequencing method based on CRISPR/Cas9 and PacBio single molecule sequencing. The 24 silkworm *FibH* sequences obtained by CACS were translated continuously without frameshift mutations. The 545 high-resolution pan-genomes were derived using whole-genome sequencing (NGS and ONT sequencing). Among the *FibH* sequences we extracted, some had polyguanine and polyN (N representing any base), as well as single-base variations that led to premature termination of translation. CEO is expensive, and the enrichment factor is affected by Cpf1/crRNA activity and chromosomal fragments, which are suitable for target gene sequencing of a small number of samples. In contrast, CACS has a low cost and high accuracy, but the cloning cycle is long; therefore, it is suitable for obtaining target genes or fragments of interest from chromosomes. The cycle of whole-genome sequencing is short, and the cost and accuracy are related to the sequencing depth; as a result, it is more appropriate for an unknown genome or mass resequencing.

We obtained a total of 286 silkworm (27 wild silkworms, 146 local, and 113 improved strains) *FibH* sequences combined this and previous study [11]. The optimal *FibH* (Opti-*FibH*) gene, which has a length of 15,960 base pairs, was found in 24.48% (70/286) of silkworms, including Family I. The maximum strength of silk is positively correlated with the length of the *FibH* gene. The longer the *FibH*, the stronger the silk fiber [11]. However, domesticated silkworms are protected by humans and do not need silk as strong as wild silkworms; the *FibH* gene is too short, silk is weak, and silk yield is low [11], which is in contrast to domestication and breeding. In addition to Opti-*FibH*, there were many *FibH* genes with similar lengths, but none of them were preferentially selected. We speculated that Opti-*FibH* may be more conducive to spinning and textile processing.

With *FibH* gene as marker gene, all silkworms were divided into four large families, and each family had at least six members containing local and improved strains. Except for these four families, the rest had few members and only local strains. Moreover, we obtained the *FibH* sequences of four heterozygotes (L185, L194, P21, and P25) from the pan-genome. The *FibH* genes of L185 and L194 were different from those of other strains. P25 had one *FibH* gene (15,315 bp) belonging to Family I, while the other was different from other *FibH* genes. The two *FibH* genes (15,960 bp and 15,315 bp) of P21 belonged to Families I and III, respectively, indicating that P21 was associated with these two families, but we could not determine its parents. More written records and genome-wide analyses are needed to identify the parents of the silkworms.

In conclusion, we globally characterized hundreds of silkworm *FibH* sequences from the pan-genome, revealed the relationship between the cis-regulatory element and silk yield, and established a silkworm pedigree. This study provides a theoretical foundation for silk improvement and silkworm breeding.

## Figures and Tables

**Table 1:** Silkworm pedigree with the same *FibH* gene.

Family	Sample ID	Sample	*FibH* Gene (bp)
Family I	L79, L90, L102, L109, L113, L172, L173, L174, L176, L178, L182, L183, L184, L200, L214, P20, P22, P35, P38, P46, P47, P49, P50, P51, P53, P54, P55, P59, P63, P64, P68, P70, P72, P73, P97, P103, P113, P119, P121, P125, P150, P153, P155, P157, P158, P161, P168, P169, P171, P172, P174, P175, P180, P183, P184, P185, P186, P195, P196, P197, P199, P208	62	15,960
Family II	L87, L94, L97, L105, L201, L208, P99, P101, P104, P105, P107, P108, P111, P114, P122, P124, P129, P131, P149, P156, P167, P176, P177, P178, P181, P187, P188, P191, P192, P193, P194	31	15,318
Family III	L47, L71, L75, L92, L98, L179, L180, L193, P18, P26, P27, P33, P34, P41, P163, P202	16	15,315
Family IV	L63, P37, P67, P74, P76, P79	6	14,874

## Data Availability

Not applicable.

## Supplementary Materials

The following are available online at https://www.mdpi.com/article/10.3390/insects14030244/s1, Table S1: The silkworm strains in detail; Table S2: The *FibH* gene sequences from silkworm pan-genome; Table S3: Sequence and length of all repetitive cores; Table S4: Differential sequence between *FibH* genes.
